# The State of the Art of Pediatric Multiple Sclerosis

**DOI:** 10.3390/ijms24098251

**Published:** 2023-05-04

**Authors:** Raluca Ioana Teleanu, Adelina-Gabriela Niculescu, Oana Aurelia Vladacenco, Eugenia Roza, Radu-Stefan Perjoc, Daniel Mihai Teleanu

**Affiliations:** 1 “Carol Davila” University of Medicine and Pharmacy, 020021 Bucharest, Romania; raluca.teleanu@umfcd.ro (R.I.T.); eugenia.roza@umfcd.ro (E.R.); radu-stefan.perjoc@rez.umfcd.ro (R.-S.P.); daniel.teleanu@umfcd.ro (D.M.T.); 2 Department of Pediatric Neurology, “Dr. Victor Gomoiu” Children’s Hospital, 022102 Bucharest, Romania; 3 Research Institute of the University of Bucharest—ICUB, University of Bucharest, 050657 Bucharest, Romania; adelina.niculescu@upb.ro; 4 Department of Science and Engineering of Oxide Materials and Nanomaterials, Politehnica University of Bucharest, 011061 Bucharest, Romania; 5 Department of Neurosurgery, Emergency University Hospital, 050098 Bucharest, Romania

**Keywords:** pediatric multiple sclerosis, multiple sclerosis treatment, emerging therapeutic strategies, clinical trials

## Abstract

Multiple sclerosis (MS) represents a chronic immune-mediated neurodegenerative disease of the central nervous system that generally debuts around the age of 20–30 years. Still, in recent years, MS has been increasingly recognized among the pediatric population, being characterized by several peculiar features compared to adult-onset disease. Unfortunately, the etiology and disease mechanisms are poorly understood, rendering the already limited MS treatment options with uncertain efficacy and safety in pediatric patients. Thus, this review aims to shed some light on the progress in MS therapeutic strategies specifically addressed to children and adolescents. In this regard, the present paper briefly discusses the etiology, risk factors, comorbidities, and diagnosis possibilities for pediatric-onset MS (POMS), further moving to a detailed presentation of current treatment strategies, recent clinical trials, and emerging alternatives. Particularly, promising care solutions are indicated, including new treatment formulations, stem cell therapies, and cognitive training methods.

## 1. Introduction

Multiple sclerosis (MS) is a chronic autoimmune-mediated neurodegenerative disease of the central nervous system (CNS). MS is the most common non-traumatic disabling disease amongst young adults and is characterized by inflammatory demyelination with axonal transection [1,2,3,4,5]. The mean age of MS onset is 20–30 years, but in recent years, the disease has been increasingly recognized in the pediatric population [2,5]. Pediatric-onset multiple sclerosis (POMS) is defined as MS with onset before the age of 18. POMS accounts for 2 to 10% of total MS cases and has the highest incidence rates between 13 and 16 years of age. In what concerns gender predisposition, MS occurs almost equally between genders before puberty, while post-puberty girls are more likely to be affected [1,6,7,8].

POMS is characterized by several distinctive clinical features compared to adult-onset MS. Pediatric disease is marked by a more active inflammation and a high rate of disabling relapses. However, the relapse recovery is faster and more complete compared to adults, disability progression is slower, and a longer time elapses before transitioning to secondary progression. Despite the better regeneration capacity, given the young manifestation age, POMS patients reach a comparable level of handicap 10 years earlier than patients with adult-onset MS (AOMS) [6,9]. Moreover, POMS can impact children’s cognitive function and development; more than 50% of MS pediatric patients continue to accrue cognitive deficits within the first five years after disease onset [8,10].

Additionally, POMS is associated with challenges in ensuring a prompt diagnosis and choosing the best treatment option [11]. Despite the existence of several diagnostic criteria for distinguishing pediatric MS from other acquired demyelinating syndromes [12,13], more in-depth research is still needed to comprehend disease particularities. Moreover, even after the correct diagnosis is decided, there is a limited range of treatment possibilities for pediatric patients, with most of the therapeutic strategies only being approved for AOMS [14].

In this context, this review aims to present the state of the art of pediatric MS, setting an up-to-date framework of the disease and serving as an inception point for future research in the field. Specifically, a brief overview of etiology, risk factors, comorbidities, and diagnosis possibilities are established before moving to a more extensive presentation of treatment strategies, including conventional treatment approaches, clinical trials, and emerging solutions (i.e., new treatment formulations, stem cell therapies, and cognitive training methods).

## 2. Etiology, Risk Factors, and Comorbidities

The etiology of MS has not yet been precisely elucidated. Nonetheless, studies hint that genetic and environmental factors contribute to increased susceptibility to developing POMS, making it a multifactorial disease [1,2,7].

More than 200 genes have been identified as susceptibility sources for adult MS, counting at least 13 major histocompatibility complex (MHC) loci. Among them, around one-third have also been linked with POMS, indicating a shared genetic inheritance. The most significant genetic contribution is associated with changes in the human leukocyte antigen (HLA) in general and the HLA-DRB1 gene in particular [2,12,15,16].

**Figure 1:**
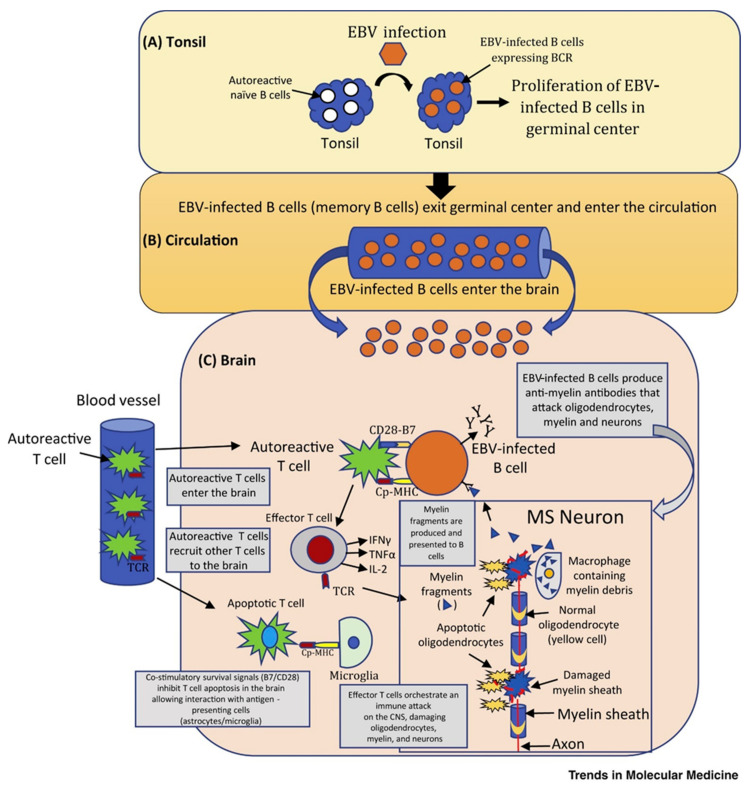
The autoreactive B cell hypothesis. Reprinted from an open-access source [20]. Abbreviations: BCR—B cell receptor; B7—co-stimulatory molecule; CD28—T cell surface receptor; CNS—central nervous system; Cp-MHC—CNS peptides bound to MHC molecules; EBV—Epstein–Barr virus; IFN—interferon; IL—interleukin; MS—multiple sclerosis; TCR—T cell receptor; TNF—tumor necrosis factor.

In addition to the genetic background, several environmental factors were also noted to influence the development of MS. One of the most studied POMS determinants is infection with Epstein–Barr virus (EBV) [1,2,4,7,12,17,18,19,20]. Studies have demonstrated a reciprocate relation [19]: the risk of developing MS increases after EBV infection [21,22], and EBV infection is more prevalent in patients with MS [23,24]. To be more specific, MS patients present elevated EBV-specific antibody levels, elevated EBV-specific CD8+ T cell responses are observed in active MS, and EBV antigens have been found in the brain tissue of MS patients, indicating viral replication [18]. According to the autoreactive B cell hypothesis, inadequate elimination of EBV-infected B cells by cytotoxic CD8^+^ T cells leads to the accumulation of EBV-infected autoreactive B cells in lymphoid tissues in the MS brain, causing prolonged exposure to local antigens [20]. The detailed mechanism is schematically represented in Figure 1.

Another important environmental factor is smoking. Whether in the form of cigarette smoking during childhood or second-hand smoking, cigarette smoke exposure was correlated with an increased risk of MS [2,4,7,10]. Particularly, parental smoking at home combined with HLA-DRB1*1501-positive status raises the risk of POMS by 3.7 times compared with monophasic acquired demyelinating syndromes (ADSs) [15].

Other risk factors include vitamin D deficiency, increased body mass index and obesity, lack of infant breastfeeding, pesticide-related exposures, air quality, and hormonal influence [1,2,4,7]. In addition, some risk factors appear to have a more significant impact during a specific time period. For example, body mass index (BMI) and obesity during adolescence, rather than childhood, are linked with a greater risk of developing MS [25,26,27,28,29]. However, none of these environmental factors or identified genetic susceptibility variants are enough to cause the disease independently. MS onset is rather the result of a “perfect storm”-type combination of multiple risk factors that produce inflammation in the CNS and dysregulate autoimmunity [20].

MS is associated with numerous comorbidities, including cardiovascular disease, chronic lung diseases, cancer, autoimmune diseases, and metabolic disorders [30,31,32,33]. Compared to AOMS, children have a lower disability risk within the first 10 years from diagnosis and a longer time lapse to the secondary progressive phase. However, POMS patients reach disability milestones at younger ages [6]. This may be caused by the fact that CNS inflammation occurs early in life when multiple organs are actively maturing [34].

Moreover, POMS patients face a significant risk of neurological disturbances and psychiatric comorbidities. Some of the most frequent such conditions are epilepsy, migraine, restless leg syndrome, fatigue, depression, anxiety, and bipolar disorder. These comorbidities pose a considerable burden on pediatric patients, reducing their quality of life and impacting their education [6,31,32,35,36,37,38].

In patients with POMS, another critical feature is represented by cognitive impairment, given that between 30 and 50% of children with MS present at least a mild cognitive deficit [37,39,40]. The affected cognitive domains include episodic memory, altered attention, visual-motor integration, processing speed, and executive functions. These aspects represent a particular concern due to how they may interfere with educational and occupational achievements [37,39,41].

There are multiple factors linked to cognitive decline in patients with POMS. Several studies revealed a correlation between grey matter atrophy, particularly in the thalamus, and cognitive decline [42,43,44,45,46,47,48,49]. MRI studies found that POMS is associated with reduced global brain and thalamic volumes when compared with healthy subjects, suggesting an early neurodegenerative process in the course of the disease [42,44,45]. Moreover, patients with POMS have reduced hippocampal and amygdala volume, regions often associated with memory and learning [47,48]. Grey matter lesions are also linked with cognitive decline in patients with MS [50,51,52,53]. Besides focal cortical damage and reduced grey matter volume, several other mechanisms have been described in cognitive impairment, such as mitochondrial resilience, alteration in metabolic pathways, and synaptic GABAergic and glutamatergic transmission. Furthermore, clinical manifestations of the disease, such as fatigue, sleep disturbances, motor impairment, and sphincter dysfunctions, can lead to school absenteeism which in turn adds to the cognitive impairment. Recent studies have found various possible biomarkers to predict POMS’s cognitive decline. Among them, the most promising ones appear to be Vitamin D deficiency and CSF-light neurofilaments [54,55].

Thus, increasing awareness and improving the understanding of comorbidities in POMS patients are essential conditions for optimizing the treatment for this population and upgrading the quality of life of the diseased children.

## 3. Diagnosis

**Figure 2:**
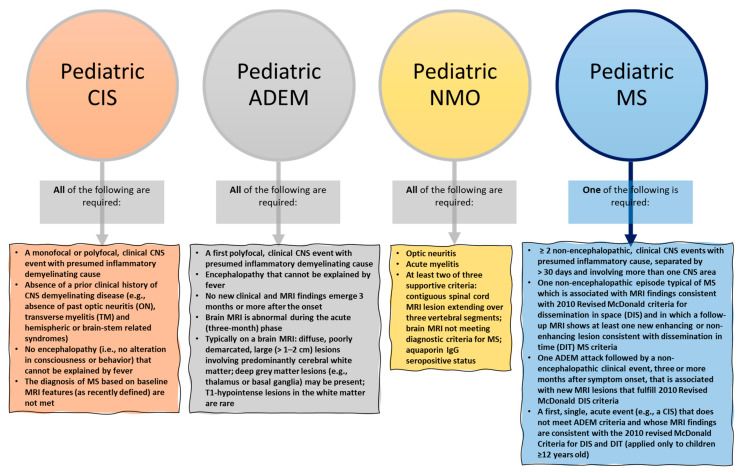
Diagnosis criteria for acquired demyelinating syndromes (ADSs). Created based on information from [12,58]. Abbreviations: ADEM—acute disseminated encephalomyelitis; CIS—clinically isolated syndrome; CNS—central nervous system; IgG—immunoglobulin G; MRI—magnetic resonance imaging; MS—multiple sclerosis; NMO—neuromyelitis optics.

The diagnosis of POMS can be challenging as this disease resembles, to some extent, a wide range of disorders of both inflammatory and non-inflammatory etiologies. Given the numerous possible MS mimics and ADS phenotypes, it is not uncommon for doctors to suppose a different diagnosis than POMS in children with acute neurologic symptoms and white matter lesions on MRI [14,56,57]. To distinguish POMS from various demyelinating syndromes that can occur in childhood, the Pediatric International Study Group established a series of criteria that have been further used in most studies [12,13] (Figure 2).

In addition to the above-mentioned criteria, cerebrospinal fluid (CSF) analysis can aid in diagnosing POMS, as it can indicate chronic CNS inflammation. Results may also reveal abnormalities in effector and regulatory T cell subsets and immune senescence. CNS-directed antibodies may be documented as well; however, their pathophysiological importance remains unclear. In contrast, the presence of serum and/or CSF antibodies recognizing aquaporin-4 (AQP4) supports the diagnosis of an NMO, distinct from MS [34]. Furthermore, the presence of anti–myelin oligodendrocyte glycoprotein (MOG) antibodies registered with similar cell-based assays can also be linked with distinct disease phenotypes in pediatric patients [34]. Despite the existence of clinical phenotypic overlaps between MOG-associated disease (MOGAD), MS, and AQP4 antibody-associated NMOSD, cumulative biological, clinical, and pathological evidence discriminates between these conditions. Specifically, MS or NMOSD diagnosis should not be attributed to patients with anti-MOG antibodies in their serum, while an ADEM diagnosis would be more plausible [59,60]. In the past, only patients who were seropositive for AQP4-immunoglobulin G were included in the NMOSD. However, several publications reported the detection of serum anti-MOG antibodies in AQP4 antibody-negative NMOSD patients, leading to the decision of the 2015 international consensus diagnostic criteria for NMOSD to include AQP4-IgG seronegative patients as well [61,62,63].

Furthermore, the presence of CSF-specific oligoclonal bands represents another criterion to help distinguish between various demyelinating syndromes. In more detail, CSF-specific oligoclonal bands are rare in MOGAD and ADEM but frequent in MS. This allows the attribution of MS diagnosis even if MRI findings on the baseline scan do not meet the criteria for DIT. Nonetheless, McDonald 2017 criteria for MS recommends caution in diagnosing children with an onset below 11 years of age and in those with encephalopathy [60,64,65].

Additionally, it has been reported that high levels of proinflammatory cytokines in the CSF and serum of MS patients can change blood–brain barrier permeability, stimulating T-lymphocyte migration into the brain and promoting disease progression [66]. Hence, cytokine detection holds promise as an alternative for the timely diagnosis of MS patients, also allowing early recognition of relapsing patients and prediction of anti-inflammatory therapy failure [3]. A useful complementary analysis is matrix metalloproteinase-9 (MMP-9) detection, as elevated levels of this MMP have been correlated with ongoing neuroinflammation processes characteristic of MS relapse [67,68].

An interesting proposal for extending MS diagnosis criteria is offered by Nikolic et al. [13]. The researchers recommend that visual evoked potentials (VEP) should be used to analyze the visual system function in POMS in regular clinical trials as an objective, fast, accessible, and inexpensive diagnostic method for detecting clinical and subclinical lesions. The addition of this analysis would improve the diagnosis of clinically silent lesions of the visual pathway, being a more sensitive method than MRI and optical coherence tomography. Recently, it has been suggested that the use of several miRNAs (i.e., miR-320a, miR-125a-5p, miR-652-3p, miR-185-5p, miR-942-5p, and miR-25-3p) as circulating biomarkers can aid diagnosing MS. Specifically, these miRNAs have been noticed to be significantly upregulated in both pediatric and adult MS patients [69].

Some other biomarkers have also been considered useful tools for the early detection of MS. For instance, serum neurofilament light chain (sNfL) has been proposed as a biomarker for monitoring disease activity and treatment response in POMS. sNfL helps predict disease severity and guide treatment decisions in MS pediatric patients, being a promising biomarker for choosing personalized therapeutic strategies [70,71]. Neurofilament heavy chain (NfH) has also been studied in relation to MS, being increased in patients with ongoing relapses. Nonetheless, when comparing NfH with sNfL, the latter discriminates better between MS and controls [72]. Additionally, glial fibrillary acidic protein (GFAP) levels were noticed to be higher in the serum of NMO spectrum disorder patients than in healthy controls and MS patients. Specifically, researchers reported that a higher sGFAP/sNfL quotient at relapse could discriminate NMOSD from MS with a sensitivity of 73.0% and a specificity of 75.8% [73].

Nonetheless, additional studies should be performed to elucidate the pathophysiologic mechanisms underlying the broad range of pediatric-onset CNS demyelinating diseases, especially those that may discern POMS from other conditions. A better understanding of disease particularities would allow for improved diagnostic modalities and more informed therapeutic decisions [34].

## 4. Treatment

It is of critical importance to correctly establish the diagnosis due to further implications in selecting the best-choice treatment. Once it is decided that the child has MS, certain therapeutic options can be tackled. However, there is a poor understanding of what concerns the safety and generalizability of disease-modifying therapy (DMT) in pediatric patients. Thus, additional challenges are faced when treating children and adolescents in comparison to adults with MS [56,57].

Moreover, given that children almost always have a relapsing-remitting form of MS, therapeutic approaches should combine treatment of relapses with immunomodulatory and symptomatic treatment. In addition, the complex nature of the disease recalls for multidisciplinary treatment, involving the expertise of pediatric neurologists, pediatricians, ophthalmologists, psychologists, physiotherapists, and, if necessary, pediatric psychiatrists and pharmacologists [2].

In this context, the following sections aim to shed some light on the current and emerging treatment strategies, counting conventional DMTs, undergoing clinical trials, and several novel approaches.

### 4.1. Conventional Approaches

Even though it has been recognized that POMS has a more prominent disease activity, earlier age at onset of disability milestones, and more prominent cognitive impairment compared with physical disability earlier in the disease course than AOMS, the conventional treatment approaches suppose the use of the same therapeutics as for adults, despite not being formally approved [14]. MS therapy is generally based on DMTs that are classified into two categories: first-line (e.g., interferon beta-1a, interferon beta-1b, and glatiramer acetate) and second-line immunomodulatory therapy (e.g., natalizumab, mitoxantrone, fingolimod, teriflunomide, azathioprine, rituximab, dimethyl fumarate, and daclizumab) [2,74,75,76] (Table 1). In addition, for the treatment of relapses, high intravenous doses of corticosteroids, intravenous immunoglobulins, and plasmapheresis can be utilized [2].

Immunomodulatory drugs have been reported to considerably diminish the frequency and severity of clinical relapses, disease activity, and degree of disability [5,74]. Nonetheless, 30% of pediatric patients with MS are partially responsive or nonresponsive to first-line therapy and discontinue this type of treatment. Despite being generally well-tolerated, these therapies may raise difficulties in pediatric patients, including side effects, toxicity, persisting relapses, and intolerance or non-adherence. When proven inefficient, first-line therapy is often switched to second-line treatment. Another possibility is to use escalation strategies that have been reported beneficial in other autoimmune disorders and may also be successful in MS [74].

Even though now more medications are available than ever, there is an increased treatment complexity and elevated concern for serious adverse effects [15]. For instance, daclizumab has been proven to be efficacious in clinical trials and used right away for MS treatment, including off-label administration in pediatric patients. In 2016, the drug was approved by the FDA and EMA as a therapeutic agent for relapsing forms of MS. However, numerous severe adverse effects were reported after market authorization, including severe inflammatory brain disease cases with fatal outcomes. These aspects resulted in the withdrawal of approval of daclizumab in Europe and the US in 2018 [14,77,78]. Safety concerns have also been raised by alemtuzumab, a drug approved as an escalation therapy for patients with MS. Despite its high efficiency in reducing relapses and brain volume loss, alemtuzumab use was correlated with numerous adverse effects and development of secondary autoimmune disease within the follow-up period, with a peak within 18–36 months from the first infusion [78,79,80].

This context highlights the difficulty of detecting rare adverse events and determining long-term effects, thus emphasizing the need for phase 4 clinical studies that monitor newly approved treatments. Additionally, full transparency of pharmaceutical companies and clinicians is vital for managing any concerns as rapidly as possible [78]. Moreover, the inherent potential differences between adults and children render DMTs uncertain regarding safety and efficiency for treating POMS [14].

### 4.2. Clinical Trials

As aforementioned, a series of DMTs were proven effective in adult patients with MS. Nonetheless, very little is known about how pediatric patients would respond to these treatments, given that, until recently, there have been no randomized controlled clinical trials or safety studies in children with MS. Fortunately, a considerably increased interest was noted in the field in the last few years, resulting in the launching of pediatric studies for assessing the effects of new drugs [9,74].

In this context, Table 2 was created to summarize information concerning all the interventional studies on pediatric multiple sclerosis retrieved from ClinicalTrails.gov, excluding the terminated and withdrawn studies.

Out of the identified clinical trials, two represent phase 1 studies, one represents phase 2, and eight represent phase 3, and this classification is not applicable to eight. Given that several treatment strategies have reached such an advanced testing stage, it can be expected that they will yield new therapeutic alternatives on the market if the results continue to be favorable.

Regarding status, there are seven completed studies, six are recruiting, two are not yet recruiting, three are active but not recruiting, and one is unknown. Among the completed clinical trials, three studies also have publicly available results, and two of them have been discussed in several articles as well. In what concerns study NCT02234713 [86], it has been reported that medication adherence depends on the ability of patients to maintain healthy habits, which is influenced by remembering/forgetting to take medicine, experiences with fatigue, and experiences with medication [100]. Researchers used an electronic monitoring device (EM) and a motivational interviewing (MI) intervention to enhance adherence to DMT. Study participants experienced worsening objective adherence measures and increased parents’ involvement in their care. However, improvements were noted in some self- and parent-reported measures, including quality of life and self-efficacy. Unfortunately, well-being was reported to be worse when exposed to MI [101].

Throughout study NCT02410200 [97], researchers investigated the safety, efficacy, and pharmacokinetics of dimethyl fumarate in POMS patients. Promising results were obtained as a significant reduction in T2 hyperintense lesion incidence was noted from baseline to the final eight weeks of treatment, adverse effects and pharmacokinetic parameters were consistent with adult findings, and no serious adverse events were reported correlated with dimethyl fumarate administration. Thus, it was concluded that this is a safe and effective treatment for POMS [102].

The undergoing clinical studies have the potential to extend current therapeutic options for patients with POMS, increasing treatment tolerance and efficacy [10]. Moreover, the existence of clinical trials focused on monitoring neurodegenerative processes, medication compliance, cognitive remediation, and exercise training will eventually lead to a global strategy toward mitigating the long-term consequences of MS.

### 4.3. Emerging Strategies

In addition to the appealing strategies that have reached the clinical trials testing stage, several novel approaches have been recently proposed as promising alternatives or adjuvants for improving the therapeutic care of POMS patients. In this respect, the following subsections discuss several new drug formulations, stem cell therapies, and cognitive training methods.

#### 4.3.1. New Treatment Formulations

Several emerging drug therapies are currently under investigation for various forms of multiple sclerosis, including Epstein–Barr virus T cell technology platforms, Bruton’s tyrosine kinase inhibitors, remyelinating strategies, immune suppressants, drugs for the humoral immune system, immune tolerance, neural protection and antioxidation [103]. Nonetheless, undergoing clinical trials for testing these approaches have not yet been addressed in pediatric patients.

One of the most attractive therapeutic strategies is to treat MS by effectively controlling EBV infection. This could be achieved by B cell depletion, administration of antiviral drugs, increasing overall immunity, or enhancing immune surveillance. Additionally, developing a vaccine against EBV might significantly contribute to MS onset reduction. However, providing sterile immunity against any herpes virus is highly challenging. Nonetheless, recent studies on other herpesvirus vaccines have reported promising outcomes, supporting the concept that designing a vaccine for preventing the disease rather than infection is a more convenient approach [20]. Alternatively, autologous EBV-specific T cell therapy was found promising as well. The MS patients treated in such a manner presented no serious adverse events and displayed some degree of clinical improvement that can be sustained for up to three years after treatment [104].

Targeting certain tetraspanins involved in regulating the cell-mediated immune response is a different possibility. For instance, CD81 is involved in lymphocyte cell membrane organization and is recognized as a major modulator of virus entry into cells. CD81 is mainly studied in the context of the hepatitis C virus, but it has been reported to play important roles in other pathogenic human viruses [105,106]. Thus, blocking CD81 may be a solution for limiting EBV entry, consequently reducing the risk of developing MS. In addition, TSPAN32 may also be involved in the pathogenesis of MS due to its immune-regulatory role [107,108]. Therefore, future research should exploit its implication in cellular immune responses for developing new therapies for immunoinflammatory/autoimmune diseases, including POMS.

Recent evidence also indicates that B cells play an essential role in MS pathogenesis through several mechanisms (e.g., antibody production, antigen presentation, T cell stimulation and activation, production of pro-inflammatory cytokines, and formation of ectopic meningeal germinal centers). The recent interest in B cells’ role in MS is mainly attributed to the profound anti-inflammatory effects of rituximab, a chimeric monoclonal antibody (mAb) targeting the B cell surface marker CD20. Given the successful results of this drug, similar selective B cell-depleting therapies may expand the treatment possibilities, especially for relapsing and progressive MS patients. Other anti-B lymphocyte monoclonal antibody formulations (i.e., ocrelizumab, ofatumumab, and ublituximab) are either in use or are being developed for the treatment of MS as well. However, future studies should focus on optimizing administration regimens for such in-use therapeutics and unveiling long-term risks [109,110,111,112].

Several remyelinating strategies can be employed to restore the deficits caused by MS demyelination, including overcoming inhibitory signals, stimulating oligodendrocyte precursor cell differentiation, or providing cofactors for myelin-forming enzymes [103]. In this respect, investigations were conducted on various therapeutics with different levels of success. Clinical trials have investigated the use of opicinumab [113], elezanumab [114], monoclonal recombinant human antibody IgM22 [115], and high-dose biotin (MD1003) [116]. Moreover, several small molecules with indications other than MS are being repurposed to promote myelin repair. Specifically, recent studies explored the value of therapeutic agents, such as bexarotene (not recommended because of its poor tolerability and negative primary outcome) [117], domperidone (35% of patients experienced considerable worsening of disability and 84% presented adverse events) [118], and clemastine fumarate (the primary efficacy endpoint was met and myelin repair could be attained even for prolonged damage) [119].

Another interesting perspective is to focus on neurotransmitter abnormalities to prevent cognitive impairment. Particularly, patients with relapsing-remitting MS (RRMS) were reported to have lower levels of GABA+ in the posterior cingulate cortex and left hippocampus than controls. Moreover, defective GABAergic interneurons would alter inhibitory signaling within cortical circuits, leading to a loss or deterioration in cognitive function. Therefore, these aspects should be considered when designing high-performing MS medication [120,121,122].

#### 4.3.2. Stem Cell Therapies

In addition to DMTs, stem-cell-based therapies have started to gain ground as well. Such cell-based therapies can lead to the regeneration of different cell types, immune response modulation, and repair stimulation [14,123,124]. Additionally, eradicating memory cells in MS through intensive immunosuppression with repopulating naïve stem cells may generate sustained remission in relapsing MS [103]. Moreover, cell replacement therapy that points to overcoming neuronal cell loss and remyelination failure is a promising therapeutic alternative for increasing endogenous myelin repair [125].

Given the potential of stem cell therapies, numerous preclinical studies have been performed on experimental autoimmune encephalomyelitis MS models, demonstrating that grafted cells with various origins (e.g., mesenchymal stem cells (MSCs), neural precursor and stem cells, and induced pluripotent stem cells) can repair CNS lesions and recover functional neurological deficits. Furthermore, studies carried out on peripherally administered autologous hematopoietic stem cells (AHSC) indicated the feasibility of cell replacement therapy for MS immunomodulatory treatment [125]. Transplantation of AHSC after immunoablation can “reset” the immune system, depleting the current autoreactive one and reconstituting a more balanced immunoregulatory framework. Such practices have reportedly been demonstrated effective in generating a sustained effect in disease activity and progression of RRMS and secondary progressive MS (SPMS). Nonetheless, the procedure has not been clinically tested in pediatric patients [14]. However, certain guiding observations can be gathered from the undergoing trials on the adult population [103]. Some of these studies are observational (NCT04674280 [126] and NCT05029206 [127]) or single arm with safety measures being the primary outcome (NCT03113162 [128], NCT00716066 [129], and NCT04203017 [130]). One phase 3 randomized interventional study of RRMS (NCT03477500) aims to compare two treatment arms, namely AHSC transplantation (AHSCT) versus drug administration (i.e., alemtuzumab, cladribine or ocrelizumab) [131], while another undergoing phase 3 study (NCT04047628) focuses on the comparison between AHSCT and Best Available Therapy (BAT) for treatment-resistant relapsing MS [132]. These studies are expected to be completed in the following years when they will hopefully provide clearly interpretable results.

MSCs represent another highly promising candidate cell population for designing MS treatment strategies. These stem cells have a high degree of plasticity and a broad range of immunomodulatory, repair, and neuroprotective properties. MSC transplantation via intravenous or intrathecal routes demonstrated feasibility, safety, and tolerability with no serious adverse reactions in AOMS patients, but no cases or trials were reported in POMS [14]. Moreover, undergoing clinical trials may further deepen the knowledge concerning MSC transplantation as a treatment modality for MS patients [103]. Specifically, several uncontrolled, single arm clinical studies utilize MSC administration as a neuroregenerative or neuroprotective treatment in MS (NCT05003388 [133], NCT03822858 [134], NCT04956744 [135], and NCT04943289 [136]), one phase 1/2 trial (NCT04749667 [137]) uses a cross-over study design to assess evoked potentials in progressive MS for investigating neuroregenerative efficacy of autologous MSC treatment, while a phase 2, randomized, blinded, placebo-controlled trial (NCT05116540 [138]) aims to establish the efficacy and safety of autologous Hope Biosciences adipose-derived mesenchymal stem cells (HB-adMSCs) administration.

Expectedly, after these studies provide confirmation and validation of stem cell transplantation potential as an advanced AOMS treatment, further clinical tests will also consider the pediatric population.

#### 4.3.3. Cognitive Training

Increasing research interest has been directed to cognitive training methods to surpass the eventuality of cognitive impairment in pediatric MS patients. One particularly appealing intervention is computerized cognitive training (CCT), which focuses on the repeated practice of controlled learning events over structured sessions, targeting specific cognitive processes rather than explicit learning. CCT usually implies game-like computerized exercises that can be adapted to individual performance. Thus, it is an inexpensive method that can be used either as a standalone intervention or as a tool in a more complex rehabilitation program, being efficacious in the cognition and behavior of various clinical populations. Nonetheless, its effects differ across populations, cognitive domains, and specific intervention design factors [139]. Thus, its utility should not be generalized before being tested on the target patients’ group.

In this respect, Simone et al. [140] have performed a pilot double-blind, randomized clinical trial to evaluate the efficacy of a home-based computerized program for retraining attention in two cohorts of POMS and attention deficit hyperactivity disorder (ADHD) patients. The researchers observed that the cognitive rehabilitation program improved global cognitive functioning in POMS patients but had a less pronounced transfer effect in the ADHD group. Considering the promising prospects for MS pediatric patients, the research group continued investigating this target population. More recently, the researchers demonstrated that a CCT specifically designed to exercise the attention domain is associated with clinically meaningful changes in SDMT scores in the short term. However, it has been concluded that additional studies on larger populations are required to confirm these findings’ clinical validity and ensure their applicability in the routine clinical practice setting [40].

Another interesting cognitive training method is proposed by Tacchino et al. [141]. The authors described the design of the Cognitive Training Kit (COGNI-TRAcK), an app for mobile devices that can be used at home for administering intensive, personalized cognitive rehabilitation intervention based on working memory exercises. Researchers reported an 84% rate of adherence to treatment and a good disposability-to-use as all the patients felt independent to use COGNI-TRAcK at home, and 94% of them understood the given instructions. Furthermore, the tested group found the exercises interesting, was motivated to perform well, did not perceive stress, was not bored, and felt amusement while performing the app tasks. Moreover, the scientists aim to improve the app by releasing more working memory exercises, promoting internet communication procedures for data transfer, and fostering remote control of the intervention.

A different approach has been tackled by Charvet et al. [142], who paired cognitive training exercises with remotely-supervised transcranial direct current stimulation (RS-tDCS) in adult MS patients. After 10 sessions of 20 min each, the RS-tDCS group displayed significantly greater improvement in complex attention and response variability composites than the group receiving cognitive training only, whereas measures of basic attention or standard cognitive measures did not differ between the groups. Hence, this combined telerehabilitation approach holds promise for improving outcomes in AOMS and represents an appealing strategy to be tested in pediatric patients for exploring its potential in POMS as well.

## 5. Conclusions and Future Perspectives

To conclude, undeniable progress has been made in treating MS in the past few decades due to the advances in understanding disease pathogenesis. However, the pediatric part of the population affected by this chronic autoimmune inflammatory condition has not been particularly addressed until recently. This resulted in using DMTs designed for adults as a treatment for POMS without being priorly approved, even though the two forms of disease present certain differences. To ensure the safety and efficacy of treatment interventions in pediatric patients, an important number of clinical trials have been completed lately or are still undergoing. Therefore, it can be expected that, after thoroughly testing novel therapeutics and treatment strategies, some of these well-performing approaches will enter the clinical setting to improve the health status and quality of life of MS pediatric patients.

To accelerate the advancement of clinical trials, digital technologies can be involved. Specifically, they can be leveraged to enhance participants’ access and engagement, reducing associated costs, and minimizing study complexity [143,144,145,146]. Moreover, significant data amounts are being gathered during clinical trials, such as demographic data, patient data collected from sensors and wearable devices, patient-reported outcomes, etc. Artificial intelligence tools can be employed to handle all this information in a timely and precise manner. Specifically, performant algorithms can help in data acquisition and processing, guaranteeing a good fit between participants and studies, enhancing digital data extraction and computational phenotyping, and aiding scientists in interpreting the trial results [143,144,146,147].

Additionally, future research should concentrate on elucidating the molecular and cellular mechanisms that underlie disease progression and the inhibitory mechanisms that prevent neuronal CNS repair, as they may be key for developing the next generation of treatments [103]. Moreover, comparative research should be performed to establish the molecular differences between AOMS and POMS, as it would help elaborate more specific treatments tailored to the needs of each population. Other future research directions should include a better understanding of risk factors, designing specifically tailored interventions for children and adolescents to improve compliance (e.g., social media, video games, interactive technologies for exercise and cognitive rehabilitation), and evaluating patient care as a global strategy, including cognitive, behavioral, and psychosocial well-being [148].

## Figures and Tables

**Table 1:** First-line and second-line immunomodulatory therapy. Created based on information from [2,15,74].

Immunomodulatory Therapy	Drug	Administration Route	Dose	Administration Frequency	Mechanism of Action	Side Effects
First-line	Interferon beta-1a	Intramuscular	30 μg	Once a week	Inhibits lymphocyte trafficking in CNS Enhances suppressor T cell activity Reduces proinflammatory cytokine production	Flulike reactions, elevated transaminases, depression, injection site reactions
		Subcutaneous	22–44 μg	Three times a week		
	Interferon beta-1b	Subcutaneous	250 μg	Every other day		
	Glatiramer acetate	Subcutaneous	20 mg	Once a day	Promotes Th2 cell activity Shifts toward an anti-inflammatory state	Injection site reactions
Second-line	Natalizumab	Intravenous	3–5 mg/kg	Once a month	mAb against alpha 4 integrin Prevents lymphocytes from crossing BBB	PML, infusion reaction, hepatotoxicity
	Mitoxantrone	Intravenous	10–20 mg/dose Up to a total of 200 mg	Once every three months	Reduces proliferation of lymphocytes	Cardiotoxicity, risk of cardiomyopathy, leukopenia, nausea, infections, alopecia, fatigue, and amenorrhea
	Fingolimod	Per os	0.5 mg (>40 kg) 0.25 mg (<40kg)	Once a day	Sphingosine 1-phosphate receptor modulator Leads to downregulation in LN and prevents activated lymphocytes from leaving LN	Bradycardia, macular edema, infection, lymphopenia, increased LFT
	Teriflunomide	Per os	7 and 14 mg	Once a day	Lymphocytopenia in T and B cells Disrupts pyridine synthesis	GI symptoms, alopecia, increased LFT, increase BP, peripheral neuropathy
	Azathioprine	Per os	2.5–3 mg	Once a day	Antagonizes purine metabolism	Gastrointestinal disturbances, skin rashes, liver toxicity, and cytopenia
	Rituximab	Intravenous	500–1000 mg	Every 6–12 months	mAb against CD 20 on B cells	Infusion reactions, PML (not in MS but has been seen in other conditions)
	Dimethyl fumarate	Per os	Initial dose: 120 mg Therapeutic dose: 240 mg	Twice daily	Nrf2 pathway Shift to Th2 or anti-inflammatory Cytokine profile Promotes antioxidant	Flushing, nausea, stomach upset, UTI, lymphopenia; PML has been reported
	Daclizumab	Subcutaneous	150 mg	Once a month	Selectively binds to the IL-2 receptor alpha-chain	Serious infections, gastrointestinal disturbances, depression, liver toxicity with an elevation of liver enzymes, and serious cutaneous events

**Table 2:** Summary of identified clinical trials.

ClinicalTrials.gov Identifier	Official Title	Intervention/Treatment	Phase	Status	Actual/Estimated Study Completion Date	Ref.
NCT04441229	A Prospective, Observational Study of Mobile Attentional Bias Modification Training (ABMT) in the Pediatric Multiple Sclerosis (MS) Population	Behavioral: ABMT mobile application	N/A	Completed	26 March 2021	[81]
NCT04660227	Effectiveness of Exercise Training in Pediatric-Onset Multiple Sclerosis Patients	Procedure: Exercise Training Other: Control Group	N/A	Recruiting	January 2022	[82]
NCT04782466	Physical Activity, Quality of Life and Disease Outcomes in Youth With Multiple Sclerosis: the ATOMIC (Active Teens Multiple Sclerosis) Physical Activity Research Program	Behavioral: Physical Activity (PA) Intervention Behavioral: Waitlist attention-control	N/A	Recruiting	September 2023	[83]
NCT03137602	ATOMIC (Active Teens With MultIple sClerosis) Teens: A Feasibility Study	Device: ATOMIC mobile app	N/A	Completed	30 September 2019	[84]
NCT02200718	A Phase I Study of NeuroVax™, a Novel Therapeutic TCR Peptide Vaccine for Pediatric Multiple Sclerosis	Biological: NeuroVax Biological: IFA Incomplete Freund’s Adjuvant	Phase 1	Not yet recruiting	9 November 2024	[85]
NCT02234713	Treatment Adherence in Pediatric Multiple Sclerosis	Behavioral: Motivational Interview Other: Video Attention Control	N/A	Completed	September 2016	[86]
NCT04445116	A Study of Endeavor™, a Video-Game Based Cognitive Remediation, in the Pediatric Multiple Sclerosis (MS) Population	Device: Action Video Game Treatment	N/A	Not yet recruiting	June 2024	[87]
NCT03190902	Cognitive Impairment in Pediatric Onset Multiple Sclerosis: Research of Biomarkers Predictive of Cognitive Impairment Progression	Behavioral: Attention Processing Training program (APT) Behavioral: nonspecific computer training (n-ST)	N/A	Completed	30 April 2016	[88]
NCT05123703	A Phase III Multicenter, Randomized, Double-Blind, Double-Dummy Study To Evaluate Safety And Efficacy Of Ocrelizumab In Comparison With Fingolimod In Children And Adolescents With Relapsing-Remitting Multiple Sclerosis	Drug: Ocrelizumab Other: Ocrelizumab Placebo Drug: Fingolimod Other: Fingolimod Placebo	Phase 3	Recruiting	5 November 2025	[89]
NCT02361697	Monitoring of Neurodegenerative Processes in Children With Multiple Sclerosis by Diffusion-weighed Magnetic Resonance Imaging (DTI)	Other: DTI-MRI	N/A	Unknown	December 2017	[90]
NCT01884935	A Phase 1, Multicenter, Open-Label, Single-Arm, Multiple Dose Study to Evaluate the Pharmacokinetics and Pharmacodynamics of Natalizumab in Pediatric Subjects With Relapsing Remitting Multiple Sclerosis (RMS)	Biological: Natalizumab	Phase 1	Completed	September 2014	[91]
NCT01892722	A 2 Year, Double-blind, Randomized, Multicenter, Active-controlled Core Phase to Evaluate Safety & Efficacy of Daily Fingolimod vs Weekly Interferon β-1a im in Pediatric Patients With Multiple Sclerosis and 5 Year Fingolimod Extension Phase	Drug: Interferon beta-1a Drug: Fingolimod Drug: Placebo capsule Drug: Placebo im injection	Phase 3	Recruiting	2 November 2028	[92]
NCT04926818	A 2-year Randomized, 3-arm, Double-blind, Non-inferiority Study Comparing the Efficacy and Safety of Ofatumumab and Siponimod Versus Fingolimod in Pediatric Patients With Multiple Sclerosis Followed by an Open-label Extension	Drug: Fingolimod Drug: Ofatumumab Drug: Siponimod Other: Fingolimod placebo Other: Siponimod placebo Other: Ofatumumab placebo	Phase 3	Recruiting	1 June 2029	[93]
NCT02201108	A Two Year, Multicenter, Randomized, Double-Blind, Placebo-Controlled, Parallel Group Trial to Evaluate Efficacy, Safety, Tolerability, and Pharmacokinetics of Teriflunomide Administered Orally Once Daily in Pediatric Patients With Relapsing Forms of Multiple Sclerosis Followed by an Open-Label Extension	Drug: Teriflunomide Drug: Placebo	Phase 3	Active, not recruiting	25 June 2025	[94]
NCT02555215	A Multicenter Extension Study to Determine the Long-Term Safety and Efficacy of BG00012 in Pediatric Subjects With Relapsing-Remitting Multiple Sclerosis	Drug: dimethyl fumarate	Phase 3	Completed	24 September 2018	[95]
NCT03958877	An Open-Label, Randomized, Multicenter, Active-Controlled, Parallel-Group Study to Evaluate the Safety, Tolerability, and Efficacy of BIIB017 in Pediatric Subjects Aged 10 to Less Than 18 Years for the Treatment of Relapsing-Remitting Multiple Sclerosis, With Optional Open-Label Extension	Drug: BIIB017 (peginterferon beta-1a) Drug: Interferon beta type 1a	Phase 3	Recruiting	5 November 2029	[96]
NCT02410200	Open-Label, Multicenter, Multiple-Dose Study of the Effect of BG00012 on MRI Lesions and Pharmacokinetics in Pediatric Subjects With Relapsing-Remitting Multiple Sclerosis Aged 10 to 17 Years	Drug: dimethyl fumarate	Phase 2	Completed	23 September 2016	[97]
NCT02283853	Open-Label, Randomized, Multicenter, Multiple-Dose, Active-Controlled, Parallel-Group, Efficacy and Safety Study of BG00012 in Children From 10 to Less Than 18 Years of Age With Relapsing-Remitting Multiple Sclerosis, With Optional Open-Label Extension	Drug: dimethyl fumarate Drug: Interferon β-1a	Phase 3	Active, not recruiting	8 September 2025	[98]
NCT03368664	A Multi-center, Open-label, Single-arm, Before and After Switch Study to Evaluate the Efficacy, Safety and Tolerability of Alemtuzumab in Paediatric Patients With Relapsing Remitting Multiple Sclerosis (RRMS) With Disease Activity on Prior Disease Modifying Therapy (DMT)	Drug: Alemtuzumab GZ402673 Drug: Glatiramer acetate Drug: Beta-Interferon Drug: Methylprednisolone Drug: Ranitidine Drug: Ceterizine Drug: Dexchlorpheniramine Drug: Paracetamol Drug: Acyclovir Drug: Prednisolone Drug: Diphenydramine Drug: Other H1 antagonist	Phase 3	Active, not recruiting	December 2025	[99]

N/A—not applicable.

## Data Availability

Not applicable.

## References

[1] Yan K., Balijepalli C., Desai K., Gullapalli L., Druyts E. (2020). Epidemiology of pediatric multiple sclerosis: A systematic literature review and meta-analysis. Mult. Scler. Relat. Disord..

[2] Jancic J., Nikolic B., Ivancevic N., Djuric V., Zaletel I., Stevanovic D., Peric S., van den Anker J.N., Samardzic J. (2016). Multiple Sclerosis in Pediatrics: Current Concepts and Treatment Options. Neurol. Ther..

[3] Abreu C.M., Soares-dos-Reis R., Melo P.N., Relvas J.B., Guimarães J., Sá M.J., Cruz A.P., Mendes Pinto I. (2018). Emerging Biosensing Technologies for Neuroinflammatory and Neurodegenerative Disease Diagnostics. Front. Mol. Neurosci..

[4] Dobson R., Giovannoni G. (2019). Multiple sclerosis—A review. Eur. J. Neurol..

[5] McGinley M.P., Goldschmidt C.H., Rae-Grant A.D. (2021). Diagnosis and treatment of multiple sclerosis: A review. Jama.

[6] Immovilli P., De Mitri P., Bazzurri V., Vollaro S., Morelli N., Biasucci G., Magnifico F., Marchesi E., Lombardelli M.L., Gelati L. (2022). The Impact of Highly Effective Treatment in Pediatric-Onset Multiple Sclerosis: A Case Series. Children.

[7] Pilotto S., Gencarelli J., Bova S., Gerosa L., Baroncini D., Olivotto S., Alfei E., Zaffaroni M., Suppiej A., Cocco E. (2021). Etiological research in pediatric multiple sclerosis: A tool to assess environmental exposures (PEDiatric Italian Genetic and enviRonment ExposurE Questionnaire). Mult. Scler. J. Exp. Transl. Clin..

[8] Padilha I.G., Fonseca A.P.A., Pettengill A.L.M., Fragoso D.C., Pacheco F.T., Nunes R.H., Maia A.C.M., da Rocha A.J. (2020). Pediatric multiple sclerosis: From clinical basis to imaging spectrum and differential diagnosis. Pediatr. Radiol..

[9] Huppke P., Huppke B., Ellenberger D., Rostasy K., Hummel H., Stark W., Brück W., Gärtner J. (2019). Therapy of highly active pediatric multiple sclerosis. Mult. Scler. J..

[10] Narula S., Hopkins S.E., Banwell B. (2015). Treatment of Pediatric Multiple Sclerosis. Curr. Treat. Options Neurol..

[11] Krupp L.B., Vieira M.C., Toledano H., Peneva D., Druyts E., Wu P., Boulos F.C. (2019). A Review of Available Treatments, Clinical Evidence, and Guidelines for Diagnosis and Treatment of Pediatric Multiple Sclerosis in the United States. J. Child Neurol..

[12] Alroughani R., Boyko A. (2018). Pediatric multiple sclerosis: A review. BMC Neurol..

[13] Nikolic B., Zaletel I., Ivancevic N., Rovcanin B., Pepic A., Samardzic J., Jancic J. (2022). The usefulness of visual evoked potentials in the assessment of the pediatric multiple sclerosis. Eur. J. Paediatr. Neurol..

[14] Macaron G., Feng J., Moodley M., Rensel M. (2019). Newer Treatment Approaches in Pediatric-Onset Multiple Sclerosis. Curr. Treat. Options Neurol..

[15] Langille M.M., Rutatangwa A., Francisco C. (2019). Pediatric multiple sclerosis: A review. Adv. Pediatr..

[16] Boiko A.N., Gusev E.I., Sudomoina M.A., Alekseenkov A.D., Kulakova O.G., Bikova O.V., Maslova O.I., Guseva M.R., Boiko S.Y., Guseva M.E. (2002). Association and linkage of juvenile MS with HLA-DR2(15) in Russians. Neurology.

[17] Banwell B., Krupp L., Kennedy J., Tellier R., Tenembaum S., Ness J., Belman A., Boiko A., Bykova O., Waubant E. (2007). Clinical features and viral serologies in children with multiple sclerosis: A multinational observational study. Lancet Neurol..

[18] Balfour  H.H., Schmeling D.O., Grimm-Geris J.M. (2020). The promise of a prophylactic Epstein–Barr virus vaccine. Pediatr. Res..

[19] Moreno M.A., Or-Geva N., Aftab B.T., Khanna R., Croze E., Steinman L., Han M.H. (2018). Molecular signature of Epstein-Barr virus infection in MS brain lesions. Neurol.-Neuroimmunol. Neuroinflammation.

[20] Bar-Or A., Pender M.P., Khanna R., Steinman L., Hartung H.-P., Maniar T., Croze E., Aftab B.T., Giovannoni G., Joshi M.A. (2020). Epstein–Barr virus in multiple sclerosis: Theory and emerging immunotherapies. Trends Mol. Med..

[21] Thacker E.L., Mirzaei F., Ascherio A. (2006). Infectious mononucleosis and risk for multiple sclerosis: A meta-analysis. Ann. Neurol..

[22] Nielsen T.R., Rostgaard K., Nielsen N.M., Koch-Henriksen N., Haahr S., Sørensen P.S., Hjalgrim H. (2007). Multiple sclerosis after infectious mononucleosis. Arch. Neurol..

[23] Pohl D., Krone B., Rostasy K., Kahler E., Brunner E., Lehnert M., Wagner H.J., Gärtner J., Hanefeld F. (2006). High seroprevalence of Epstein–Barr virus in children with multiple sclerosis. Neurology.

[24] Alotaibi S., Kennedy J., Tellier R., Stephens D., Banwell B. (2004). Epstein-Barr virus in pediatric multiple sclerosis. Jama.

[25] Munger K.L., Bentzen J., Laursen B., Stenager E., Koch-Henriksen N., Sørensen T.I.A., Baker J.L. (2013). Childhood body mass index and multiple sclerosis risk: A long-term cohort study. Mult. Scler. J..

[26] Hedström A.K., Olsson T., Alfredsson L. (2015). Body mass index during adolescence, rather than childhood, is critical in determining MS risk. Mult. Scler. J..

[27] Gianfrancesco M.A., Acuna B., Shen L., Briggs F.B.S., Quach H., Bellesis K.H., Bernstein A., Hedstrom A.K., Kockum I., Alfredsson L. (2014). Obesity during childhood and adolescence increases susceptibility to multiple sclerosis after accounting for established genetic and environmental risk factors. Obes. Res. Clin. Pract..

[28] Liu Z., Zhang T.T., Yu J., Liu Y.L., Qi S.F., Zhao J.J., Liu D.W., Tian Q.B. (2016). Excess Body Weight during Childhood and Adolescence Is Associated with the Risk of Multiple Sclerosis: A Meta-Analysis. Neuroepidemiology.

[29] Annette L.-G., Sonu M.B., Brandon E.B., Corinna K. (2013). Childhood obesity and risk of pediatric multiple sclerosis and clinically isolated syndrome. Neurology.

[30] Hauer L., Perneczky J., Sellner J. (2021). A global view of comorbidity in multiple sclerosis: A systematic review with a focus on regional differences, methodology, and clinical implications. J. Neurol..

[31] Nociti V., Romozzi M. (2022). Multiple Sclerosis and Autoimmune Comorbidities. J. Pers. Med..

[32] Magyari M., Sorensen P.S. (2020). Comorbidity in Multiple Sclerosis. Front. Neurol..

[33] Chavda V., Patel V., Patel S. (2021). Investigation of neuroprotective potential of anti-diabetic agents in ischemic brain proteomics through in-silico molecular simulation studies. Biointerface Res. Appl. Chem..

[34] Bar-Or A., Hintzen R.Q., Dale R.C., Rostasy K., Brück W., Chitnis T. (2016). Immunopathophysiology of pediatric CNS inflammatory demyelinating diseases. Neurology.

[35] Storm van’s Gravesande K., Blaschek A., Calabrese P., Rostásy K., Huppke P., Kessler J.J., Kalbe E., Mall V. (2019). Fatigue and depression predict health-related quality of life in patients with pediatric-onset multiple sclerosis. Mult. Scler. Relat. Disord..

[36] Yeh E.A. (2021). Real-world outcomes in pediatric MS: Psychiatric comorbidities and school performance. Mult. Scler. J..

[37] Boesen M.S., Blinkenberg M., Thygesen L.C., Eriksson F., Magyari M. (2020). School performance, psychiatric comorbidity, and healthcare utilization in pediatric multiple sclerosis: A nationwide population-based observational study. Mult. Scler. J..

[38] Marchesi O., Vizzino C., Filippi M., Rocca M.A. (2022). Current perspectives on the diagnosis and management of fatigue in multiple sclerosis. Expert Rev. Neurother..

[39] Johnen A., Elpers C., Riepl E., Landmeyer N.C., Krämer J., Polzer P., Lohmann H., Omran H., Wiendl H., Göbel K. (2019). Early effective treatment may protect from cognitive decline in paediatric multiple sclerosis. Eur. J. Paediatr. Neurol..

[40] Simone M., Viterbo R.G., Margari L., Iaffaldano P. (2021). A Randomized Computer-Assisted Rehabilitation Trial of Attention in Pediatric Multiple Sclerosis: A Post Hoc Analysis. Brain Sci..

[41] Govindarajan S.T., Pan R., Krupp L., Charvet L., Duong T.Q. (2021). Gray Matter Morphometry Correlates with Attentional Efficiency in Young-Adult Multiple Sclerosis. Brain Sci..

[42] Till C., Ghassemi R., Aubert-Broche B., Kerbrat A., Collins D.L., Narayanan S., Arnold D.L., Desrocher M., Sled J.G., Banwell B.L. (2011). MRI correlates of cognitive impairment in childhood-onset multiple sclerosis. Neuropsychology.

[43] Mesaros S., Rocca M.A., Absinta M., Ghezzi A., Milani N., Moiola L., Veggiotti P., Comi G., Filippi M. (2008). Evidence of thalamic gray matter loss in pediatric multiple sclerosis. Neurology.

[44] Kerbrat A., Aubert-Broche B., Fonov V., Narayanan S., Sled J.G., Arnold D.A., Banwell B., Collins D.L. (2012). Reduced head and brain size for age and disproportionately smaller thalami in child-onset MS. Neurology.

[45] Bérengère A.-B., Vladimir F., Sridar N., Douglas L.A., David A., Dumitru F., Christine T., John G.S., Brenda B., Collins D.L. (2014). Onset of multiple sclerosis before adulthood leads to failure of age-expected brain growth. Neurology.

[46] Till C., Deotto A., Tipu V., Sled J.G., Bethune A., Narayanan S., Arnold D.L., Banwell B.L. (2011). White matter integrity and math performance in pediatric multiple sclerosis: A diffusion tensor imaging study. Neuro Rep..

[47] Rocca M.A., Morelli M.E., Amato M.P., Moiola L., Ghezzi A., Veggiotti P., Capra R., Pagani E., Portaccio E., Fiorino A. (2016). Regional hippocampal involvement and cognitive impairment in pediatric multiple sclerosis. Mult. Scler. J..

[48] Green R., Adler A., Banwell B.L., Fabri T.L., Yeh E.A., Collins D.L., Sled J.G., Narayanan S., Till C. (2018). Involvement of the Amygdala in Memory and Psychosocial Functioning in Pediatric-Onset Multiple Sclerosis. Dev. Neuropsychol..

[49] Maria A.R., Martina A., Maria Pia A., Lucia M., Angelo G., Pierangelo V., Ruggero C., Emilio P., Agnese F., Lorena P. (2014). Posterior brain damage and cognitive impairment in pediatric multiple sclerosis. Neurology.

[50] Amato M.P., Portaccio E., Goretti B., Zipoli V., Battaglini M., Bartolozzi M.L., Stromillo M.L., Guidi L., Siracusa G., Sorbi S. (2007). Association of Neocortical Volume Changes With Cognitive Deterioration in Relapsing-Remitting Multiple Sclerosis. Arch. Neurol..

[51] Calabrese M., Agosta F., Rinaldi F., Mattisi I., Grossi P., Favaretto A., Atzori M., Bernardi V., Barachino L., Rinaldi L. (2009). Cortical Lesions and Atrophy Associated With Cognitive Impairment in Relapsing-Remitting Multiple Sclerosis. Arch. Neurol..

[52] Nelson F., Datta S., Garcia N., Rozario N.L., Perez F., Cutter G., Narayana P.A., Wolinsky J.S. (2011). Intracortical lesions by 3T magnetic resonance imaging and correlation with cognitive impairment in multiple sclerosis. Mult. Scler. J..

[53] Geurts J.J.G., Pouwels P.J.W., Uitdehaag B.M.J., Polman C.H., Barkhof F., Castelijns J.A. (2005). Intracortical lesions in multiple sclerosis: Improved detection with 3D double inversion-recovery MR imaging. Radiology.

[54] Gaetani L., Salvadori N., Lisetti V., Eusebi P., Mancini A., Gentili L., Borrelli A., Portaccio E., Sarchielli P., Blennow K. (2019). Cerebrospinal fluid neurofilament light chain tracks cognitive impairment in multiple sclerosis. J. Neurol..

[55] Compas B.E., Jaser S.S., Reeslund K., Patel N., Yarboi J. (2017). Neurocognitive deficits in children with chronic health conditions. Am. Psychol..

[56] Galardi M.M., Gaudioso C., Ahmadi S., Evans E., Gilbert L., Mar S. (2019). Differential Diagnosis of Pediatric Multiple Sclerosis. Children.

[57] Pérez C.A., Smith A., Perez C.A., Nelson F. (2021). Pediatric Multiple Sclerosis. Multiple Sclerosis: A Practical Manual for Hospital and Outpatient Care.

[58] Krupp L.B., Tardieu M., Amato M.P., Banwell B., Chitnis T., Dale R.C., Ghezzi A., Hintzen R., Kornberg A., Pohl D. (2013). International Pediatric Multiple Sclerosis Study Group criteria for pediatric multiple sclerosis and immune-mediated central nervous system demyelinating disorders: Revisions to the 2007 definitions. Mult. Scler. J..

[59] Marignier R., Hacohen Y., Cobo-Calvo A., Pröbstel A.-K., Aktas O., Alexopoulos H., Amato M.-P., Asgari N., Banwell B., Bennett J. (2021). Myelin-oligodendrocyte glycoprotein antibody-associated disease. Lancet Neurol..

[60] Boesen M.S., Langkilde A.R., Ilginiene J., Magyari M., Blinkenberg M. (2022). Oligoclonal bands, age 11–17 years, occipital lesion, and female sex differentiate pediatric multiple sclerosis from acute disseminated encephalomyelitis: A nationwide cohort study. Mult. Scler. Relat. Disord..

[61] Borisow N., Mori M., Kuwabara S., Scheel M., Paul F. (2018). Diagnosis and Treatment of NMO Spectrum Disorder and MOG-Encephalomyelitis. Front Neurol.

[62] Kunchok A., Chen J.J., Saadeh R.S., Wingerchuk D.M., Weinshenker B.G., Flanagan E.P., Pittock S.J. (2020). Application of 2015 Seronegative Neuromyelitis Optica Spectrum Disorder Diagnostic Criteria for Patients With Myelin Oligodendrocyte Glycoprotein IgG–Associated Disorders. JAMA Neurol..

[63] Dean M.W., Brenda B., Jeffrey L.B., Philippe C., William C., Tanuja C., Jérôme de S., Kazuo F., Benjamin G., Anu J. (2015). International consensus diagnostic criteria for neuromyelitis optica spectrum disorders. Neurology.

[64] Thompson A.J., Banwell B.L., Barkhof F., Carroll W.M., Coetzee T., Comi G., Correale J., Fazekas F., Filippi M., Freedman M.S. (2018). Diagnosis of multiple sclerosis: 2017 revisions of the McDonald criteria. Lancet Neurol..

[65] Boesen M.S., Born A.P., Jensen P.E.H., Sellebjerg F., Blinkenberg M., Lydolph M.C., Jørgensen M.K., Rosenberg L., Thomassen J.Q., Børresen M.L. (2019). Diagnostic Value of Oligoclonal Bands in Children: A Nationwide Population-Based Cohort Study. Pediatr. Neurol..

[66] Khaibullin T., Ivanova V., Martynova E., Cherepnev G., Khabirov F., Granatov E., Rizvanov A., Khaiboullina S. (2017). Elevated levels of proinflammatory cytokines in cerebrospinal fluid of multiple sclerosis patients. Front. Immunol..

[67] Fainardi E., Castellazzi M., Bellini T., Manfrinato M.C., Baldi E., Casetta I., Paolino E., Granieri E., Dallocchio F. (2006). Cerebrospinal fluid and serum levels and intrathecal production of active matrix metalloproteinase-9 (MMP-9) as markers of disease activity in patients with multiple sclerosis. Mult. Scler. J..

[68] Biela A., Watkinson M., Meier U.C., Baker D., Giovannoni G., Becer C.R., Krause S. (2015). Disposable MMP-9 sensor based on the degradation of peptide cross-linked hydrogel films using electrochemical impedance spectroscopy. Biosens. Bioelectron..

[69] Nuzziello N., Vilardo L., Pelucchi P., Consiglio A., Liuni S., Trojano M., Liguori M. (2018). Investigating the Role of MicroRNA and Transcription Factor Co-regulatory Networks in Multiple Sclerosis Pathogenesis. Int. J. Mol. Sci..

[70] Hyun J.-W., Kim Y., Kim G., Kim S.-H., Kim H.J. (2020). Longitudinal analysis of serum neurofilament light chain: A potential therapeutic monitoring biomarker for multiple sclerosis. Mult. Scler. J..

[71] Reinert M.-C., Benkert P., Wuerfel J., Michalak Z., Ruberte E., Barro C., Huppke P., Stark W., Kropshofer H., Tomic D. (2020). Serum neurofilament light chain is a useful biomarker in pediatric multiple sclerosis. Neurol. -Neuroimmunol. Neuroinflammation.

[72] Varhaug K.N., Torkildsen Ø., Myhr K.-M., Vedeler C.A. (2019). Neurofilament light chain as a biomarker in multiple sclerosis. Front. Neurol..

[73] Mitsuru W., Yuri N., Zuzanna M., Noriko I., Christian B., David L., Takuya M., Fumie H., Ryo Y., Jens K. (2019). Serum GFAP and neurofilament light as biomarkers of disease activity and disability in NMOSD. Neurology.

[74] Jančić J., Nikolić B., Ivančević N., Henčić B., Samardžić J., Zagon I.S., McLaughlin P.J. (2017). Multiple sclerosis therapies in pediatric patients: Challenges and opportunities. Multiple Sclerosis: Perspectives in Treatment and Pathogenesis.

[75] Goldenberg M.M. (2012). Multiple sclerosis review. P T A Peer-Rev. J. Formul. Manag..

[76] Hauser S.L., Cree B.A.C. (2020). Treatment of Multiple Sclerosis: A Review. Am. J. Med..

[77] Rommer P.S., Berger K., Ellenberger D., Fneish F., Simbrich A., Stahmann A., Zettl U.K. (2020). Management of MS Patients Treated With Daclizumab–a Case Series of 267 Patients. Front. Neurol..

[78] Lancet T. (2018). End of the road for daclizumab in multiple sclerosis. Lancet.

[79] Haghikia A., Dendrou C.A., Schneider R., Grüter T., Postert T., Matzke M., Stephanik H., Fugger L., Gold R. (2017). Severe B-cell-mediated CNS disease secondary to alemtuzumab therapy. Lancet Neurol..

[80] Tzanetakos D., Breza M., Tzartos J.S., Bontzos G., Vakrakou A.G., Dermentzoglou A., Gkizis I., Smoustopoulos G., Evangelopoulos M.-E., Stefanis L. (2022). Alemtuzumab-induced alopecia universalis and transient accommodation spasm in a patient with multiple sclerosis. Ther. Adv. Neurol. Disord..

[81] Health N.L. Mobile Attentional Bias Modification Training in Pediatric MS. https://www.clinicaltrials.gov/ct2/show/NCT04441229?type=Intr&cond=Pediatric+Multiple+Sclerosis&draw=5&rank=1.

[82] Vural P. Exercise Training in Pediatric-Onset Multiple Sclerosis Patients. https://www.clinicaltrials.gov/ct2/show/NCT04660227?type=Intr&cond=Pediatric+Multiple+Sclerosis&draw=5&rank=2.

[83] Yeh E.A. ATOMIC (Active Teens Multiple Sclerosis) Physical Activity Research Program. https://www.clinicaltrials.gov/ct2/show/NCT04782466?type=Intr&cond=Pediatric+Multiple+Sclerosis&draw=5&rank=3.

[84] Yeh E.A. ATOMIC (Active Teens with MultIple sClerosis) Teens: A Feasibility Study. https://www.clinicaltrials.gov/ct2/show/NCT03137602?type=Intr&cond=Pediatric+Multiple+Sclerosis&draw=5&rank=4.

[85] BioPharma I.R. A Study of NeuroVax™, a Novel Therapeutic TCR Peptide Vaccine for Pediatric Multiple Sclerosis. https://www.clinicaltrials.gov/ct2/show/NCT02200718?type=Intr&cond=Pediatric+Multiple+Sclerosis&draw=5&rank=5.

[86] Yeh E.A. Adherence in Pediatric Multiple Sclerosis. https://www.clinicaltrials.gov/ct2/show/NCT02234713?type=Intr&cond=Pediatric+Multiple+Sclerosis&draw=5&rank=6.

[87] Health N.L. Endeavor™ in Pediatric MS (Akili). https://www.clinicaltrials.gov/ct2/show/NCT04445116?type=Intr&cond=Pediatric+Multiple+Sclerosis&draw=5&rank=7.

[88] Iaffaldano P. Cognitive Impairment in Pediatric Onset Multiple Sclerosis. https://www.clinicaltrials.gov/ct2/show/NCT03190902?type=Intr&cond=Pediatric+Multiple+Sclerosis&draw=5&rank=9.

[89] Roche H.-L. A Study To Evaluate Safety And Efficacy of Ocrelizumab in Comparison with Fingolimod in Children and Adolescents with Relapsing-Remitting Multiple Sclerosis (Operetta 2). https://www.clinicaltrials.gov/ct2/show/NCT05123703?type=Intr&cond=Pediatric+Multiple+Sclerosis&draw=5&rank=10.

[90] Muenster U.H. DTI in Children with Multiple Sclerosis. https://www.clinicaltrials.gov/ct2/show/NCT02361697?type=Intr&cond=Pediatric+Multiple+Sclerosis&draw=5&rank=12.

[91] Biogen PK and PD Study of Natalizumab in Pediatric Subjects with RRMS. https://www.clinicaltrials.gov/ct2/show/NCT01884935?type=Intr&cond=Pediatric+Multiple+Sclerosis&draw=5&rank=13.

[92] Pharmaceuticals N. Safety and Efficacy of Fingolimod in Pediatric Patients with Multiple Sclerosis. https://www.clinicaltrials.gov/ct2/show/NCT01892722?type=Intr&cond=Pediatric+Multiple+Sclerosis&draw=5&rank=14.

[93] Pharmaceuticals N. Efficacy and Safety of Ofatumumab and Siponimod Compared to Fingolimod in Pediatric Patients with Multiple Sclerosis (NEOS). https://www.clinicaltrials.gov/ct2/show/NCT04926818?type=Intr&cond=Pediatric+Multiple+Sclerosis&draw=5&rank=15.

[94] Sanofi Efficacy, Safety and Pharmacokinetics of Teriflunomide in Pediatric Patients with Relapsing Forms of Multiple Sclerosis (TERIKIDS). https://www.clinicaltrials.gov/ct2/show/NCT02201108?type=Intr&cond=Pediatric+Multiple+Sclerosis&draw=5&rank=16.

[95] Biogen Extension Study of BG00012 in Pediatric Subjects with Relapsing Remitting Multiple Sclerosis (RRMS). https://www.clinicaltrials.gov/ct2/show/NCT02555215?type=Intr&cond=Pediatric+Multiple+Sclerosis&draw=5&rank=17.

[96] Biogen A Study to Evaluate the Safety, Tolerability, and Efficacy of BIIB017 (Peginterferon Beta-1a) in Pediatric Participants for the Treatment of Relapsing-Remitting Multiple Sclerosis. https://www.clinicaltrials.gov/ct2/show/NCT03958877?type=Intr&cond=Pediatric+Multiple+Sclerosis&draw=5&rank=19.

[97] Biogen Study of the Effect of BG00012 on MRI Lesions and Pharmacokinetics in Pediatric Subjects with RRMS (FOCUS). https://www.clinicaltrials.gov/ct2/show/NCT02410200?type=Intr&cond=Pediatric+Multiple+Sclerosis&draw=5&rank=20.

[98] Biogen Phase 3 Efficacy and Safety Study of BG00012 in Pediatric Subjects with Relapsing-Remitting Multiple Sclerosis (RRMS) (CONNECT). https://www.clinicaltrials.gov/ct2/show/NCT02283853?type=Intr&cond=Pediatric+Multiple+Sclerosis&draw=5&rank=21.

[99] Sanofi A Study to Evaluate Efficacy, Safety, and Tolerability of Alemtuzumab in Pediatric Patients with RRMS with Disease Activity on Prior DMT (LemKids). https://www.clinicaltrials.gov/ct2/show/NCT03368664?type=Intr&cond=Pediatric+Multiple+Sclerosis&draw=5&rank=22.

[100] Yeh E.A., Chiang N., Darshan B., Nejati N., Grover S.A., Schwartz C.E., Slater R., Finlayson M. (2019). Adherence in Youth with Multiple Sclerosis: A Qualitative Assessment of Habit Formation, Barriers, and Facilitators. Qual. Health Res..

[101] Yeh E.A., Grover S.A., Powell V.E., Alper G., Banwell B.L., Edwards K., Gorman M., Graves J., Lotze T.E., Mah J.K. (2017). Impact of an electronic monitoring device and behavioral feedback on adherence to multiple sclerosis therapies in youth: Results of a randomized trial. Qual. Life Res. Int. J. Qual. Life Asp. Treat. Care Rehabil..

[102] Alroughani R., Das R., Penner N., Pultz J., Taylor C., Eraly S. (2018). Safety and Efficacy of Delayed-Release Dimethyl Fumarate in Pediatric Patients With Relapsing Multiple Sclerosis (FOCUS). Pediatr. Neurol..

[103] Cree B.A.C., Hartung H.-P., Barnett M. (2022). New drugs for multiple sclerosis: New treatment algorithms. Curr. Opin. Neurol..

[104] Ioannides Z.A., Csurhes P.A., Douglas N.L., Mackenroth G., Swayne A., Thompson K.M., Hopkins T.J., Green K.A., Blum S., Hooper K.D. (2021). Sustained clinical improvement in a subset of patients with progressive multiple sclerosis treated with Epstein–Barr virus-specific T cell therapy. Front. Neurol..

[105] Becic A., Leifeld J., Shaukat J., Hollmann M. (2022). Tetraspanins as potential modulators of glutamatergic synaptic function. Front. Mol. Neurosci..

[106] Bailly C., Thuru X. (2023). Targeting of Tetraspanin CD81 with Monoclonal Antibodies and Small Molecules to Combat Cancers and Viral Diseases. Cancers.

[107] Basile M.S., Mazzon E., Mangano K., Pennisi M., Petralia M.C., Lombardo S.D., Nicoletti F., Fagone P., Cavalli E. (2020). Impaired Expression of Tetraspanin 32 (TSPAN32) in Memory T Cells of Patients with Multiple Sclerosis. Brain Sci..

[108] Lombardo S.D., Mazzon E., Basile M.S., Campo G., Corsico F., Presti M., Bramanti P., Mangano K., Petralia M.C., Nicoletti F. (2019). Modulation of Tetraspanin 32 (TSPAN32) Expression in T Cell-Mediated Immune Responses and in Multiple Sclerosis. Int. J. Mol. Sci..

[109] Milo R. (2019). Therapies for multiple sclerosis targeting B cells. Croat. Med. J..

[110] Kufukihara K. (2021). Anti-B cell therapies in multiple sclerosis. Clin. Exp. Neuroimmunol..

[111] Ancau M., Berthele A., Hemmer B. (2019). CD20 monoclonal antibodies for the treatment of multiple sclerosis: Up-to-date. Expert Opin. Biol. Ther..

[112] Margoni M., Preziosa P., Filippi M., Rocca M.A. (2022). Anti-CD20 therapies for multiple sclerosis: Current status and future perspectives. J. Neurol..

[113] Cadavid D., Mellion M., Hupperts R., Edwards K.R., Calabresi P.A., Drulović J., Giovannoni G., Hartung H.-P., Arnold D.L., Fisher E. (2019). Safety and efficacy of opicinumab in patients with relapsing multiple sclerosis (SYNERGY): A randomised, placebo-controlled, phase 2 trial. Lancet Neurol..

[114] Kalluri H.V., Rosebraugh M.R., Misko T.P., Ziemann A., Liu W., Cree B.A.C. (2023). Phase 1 Evaluation of Elezanumab (Anti–Repulsive Guidance Molecule A Monoclonal Antibody) in Healthy and Multiple Sclerosis Participants. Ann. Neurol..

[115] Eisen A., Greenberg B.M., Bowen J.D., Arnold D.L., Caggiano A.O. (2017). A double-blind, placebo-controlled, single ascending-dose study of remyelinating antibody rHIgM22 in people with multiple sclerosis. Mult. Scler. J.–Exp. Transl. Clin..

[116] Cree B.A.C., Cutter G., Wolinsky J.S., Freedman M.S., Comi G., Giovannoni G., Hartung H.P., Arnold D., Kuhle J., Block V. (2020). Safety and efficacy of MD1003 (high-dose biotin) in patients with progressive multiple sclerosis (SPI2): A randomised, double-blind, placebo-controlled, phase 3 trial. Lancet. Neurol..

[117] Brown J.W.L., Cunniffe N.G., Prados F., Kanber B., Jones J.L., Needham E., Georgieva Z., Rog D., Pearson O.R., Overell J. (2021). Safety and efficacy of bexarotene in patients with relapsing-remitting multiple sclerosis (CCMR One): A randomised, double-blind, placebo-controlled, parallel-group, phase 2a study. Lancet. Neurol..

[118] Koch M.W., Sage K., Kaur S., Kim J., Cerchiaro G., Yong V.W., Cutter G.R., Metz L.M. (2021). Repurposing Domperidone in Secondary Progressive Multiple Sclerosis. Neurology.

[119] Green A.J., Gelfand J.M., Cree B.A., Bevan C., Boscardin W.J., Mei F., Inman J., Arnow S., Devereux M., Abounasr A. (2017). Clemastine fumarate as a remyelinating therapy for multiple sclerosis (ReBUILD): A randomised, controlled, double-blind, crossover trial. Lancet.

[120] Cao G., Edden R.A.E., Gao F., Li H., Gong T., Chen W., Liu X., Wang G., Zhao B. (2018). Reduced GABA levels correlate with cognitive impairment in patients with relapsing-remitting multiple sclerosis. Eur. Radiol..

[121] Teleanu R.I., Niculescu A.-G., Roza E., Vladâcenco O., Grumezescu A.M., Teleanu D.M. (2022). Neurotransmitters-Key Factors in Neurological and Neurodegenerative Disorders of the Central Nervous System. Int. J. Mol. Sci..

[122] Syaifie P.H., Hemasita A.W., Nugroho D.W., Mardliyati E., Anshori I. (2022). In silico investigation of propolis compounds as potential neuroprotective agent. Biointerface Res. Appl. Chem..

[123] Bahardoust M. (2021). Role of adipose-derived mesenchymal stem cells in the regeneration of cardiac tissue and improvement of cardiac function: A narrative review. Biointerface Res. Appl. Chem..

[124] Hassan N.S., Shaheen N.E.M., Haggag N.Z., Hassen M.T. (2022). Mesenchymal Stem Cells and/or l-2-oxothiazolidine-4–Carboxylate Improve Hyperlipidemia and Lung Cell Proliferation in Chlorpyrifos-Treated Rats. Biointerface Res. Appl. Chem..

[125] Genc B., Bozan H.R., Genc S., Genc K. Stem Cell Therapy for Multiple Sclerosis. Proceedings of the Tissue Engineering and Regenerative Medicine.

[126] European Society for Blood and Marrow Transplantation Observational Study in Multiple Sclerosis Patients Treated with Autologous Hematopoietic Stem Cell Transplantation (OMST). https://clinicaltrials.gov/ct2/show/NCT04674280.

[127] University U. Hematopoietic Stem Cell Transplantation for Treatment of Multiple Sclerosis in Sweden (AutoMS-Swe). https://clinicaltrials.gov/ct2/show/NCT05029206.

[128] Dasig D.A. Reduced-intensity Immunoablation and Autologous Hematopoietic Stem Cell Transplantation (AHSCT) for Multiple Sclerosis. https://clinicaltrials.gov/ct2/show/NCT03113162.

[129] Center F.H.C. Autologous Peripheral Blood Stem Cell Transplant for Neurologic Autoimmune Diseases. https://clinicaltrials.gov/ct2/show/NCT00716066.

[130] Moiseev I.S. Fecal Microbiota Transplantation After Autologous HSCT in Patients with Multiple Sclerosis. https://clinicaltrials.gov/ct2/show/NCT04203017.

[131] Hospital H.U. RCT Comparing Autologous Hematopoietic Stem Cell Transplantation Versus Alemtuzumab, Cladribine or Ocrelizumab in MS (RAM-MS). https://clinicaltrials.gov/ct2/show/NCT03477500.

[132] National Institute of Allergy and Infectious Diseases (NIAID) Best Available Therapy Versus Autologous Hematopoetic Stem Cell Transplant for Multiple Sclerosis (BEAT-MS) (BEAT-MS). https://clinicaltrials.gov/ct2/show/NCT04047628.

[133] The Foundation for Orthopaedics and Regenerative Medicine Safety of Cultured Allogeneic Adult Umbilical Cord Derived Mesenchymal Stem Cell Intravenous Infusion for MS. https://clinicaltrials.gov/ct2/show/NCT05003388.

[134] Tisch Multiple Sclerosis Research Center of New York Expanded Access to Intrathecal Administration of Autologous Mesenchymal Stem Cell-derived Neural Progenitors (MSC-NP) in Progressive Multiple Sclerosis. https://clinicaltrials.gov/ct2/show/NCT03822858.

[135] Biotechnology I. A Study to Evaluate the Safety, Tolerability, and Exploratory Efficacy of IMS001 in Subjects With Multiple Sclerosis. https://clinicaltrials.gov/ct2/show/NCT04956744.

[136] Kurtzberg J. Intrathecal Administration of DUOC-01 in Adults With Primary Progressive Multiple Sclerosis (DUOC for MS). https://clinicaltrials.gov/ct2/show/NCT04943289.

[137] Hospital H.U. Study of Mesenchymal Autologous Stem Cells as Regenerative Treatment for Multiple Sclerosis (SMART-MS). https://clinicaltrials.gov/ct2/show/NCT04749667.

[138] Foundation H.B.S.C.R. Randomized Double-Blind Phase 2 Efficacy and Safety of Autologous HB-MSCs vs Placebo for Treatment of Multiple Sclerosis (HBMS01). https://clinicaltrials.gov/ct2/show/NCT05116540.

[139] Lampit A., Heine J., Finke C., Barnett M.H., Valenzuela M., Wolf A., Leung I.H.K., Hill N.T.M. (2019). Computerized cognitive training in multiple sclerosis: A systematic review and meta-analysis. Neurorehabilit. Neural Repair.

[140] Simone M., Viterbo R.G., Margari L., Iaffaldano P. (2018). Computer-assisted rehabilitation of attention in pediatric multiple sclerosis and ADHD patients: A pilot trial. BMC Neurol..

[141] Tacchino A., Pedullà L., Bonzano L., Vassallo C., Battaglia M.A., Mancardi G., Bove M., Brichetto G. (2015). A new app for at-home cognitive training: Description and pilot testing on patients with multiple sclerosis. JMIR Mhealth Uhealth.

[142] Charvet L., Shaw M., Dobbs B., Frontario A., Sherman K., Bikson M., Datta A., Krupp L., Zeinapour E., Kasschau M. (2018). Remotely Supervised Transcranial Direct Current Stimulation Increases the Benefit of At-Home Cognitive Training in Multiple Sclerosis. Neuromodulation Technol. Neural Interface.

[143] Alemayehu D., Hemmings R., Natarajan K., Roychoudhury S. (2022). Perspectives on Virtual (Remote) Clinical Trials as the “New Normal” to Accelerate Drug Development. Clin. Pharmacol. Ther..

[144] Inan O.T., Tenaerts P., Prindiville S.A., Reynolds H.R., Dizon D.S., Cooper-Arnold K., Turakhia M., Pletcher M.J., Preston K.L., Krumholz H.M. (2020). Digitizing clinical trials. npj Digit. Med..

[145] Rosa C., Marsch L.A., Winstanley E.L., Brunner M., Campbell A.N.C. (2021). Using digital technologies in clinical trials: Current and future applications. Contemp. Clin. Trials.

[146] Busnatu Ș.S., Niculescu A.-G., Bolocan A., Andronic O., Pantea Stoian A.M., Scafa-Udriște A., Stănescu A.M., Păduraru D.N., Nicolescu M.I., Grumezescu A.M. (2022). A Review of Digital Health and Biotelemetry: Modern Approaches towards Personalized Medicine and Remote Health Assessment. J. Pers. Med..

[147] Weatherall J., Khan F.M., Patel M., Dearden R., Shameer K., Dennis G., Feldberg G., White T., Khosla S., Ashenden S.K. (2021). Chapter 10—Clinical trials, real-world evidence, and digital medicine. The Era of Artificial Intelligence, Machine Learning, and Data Science in the Pharmaceutical Industry.

[148] Parrish J.B., Fields E. (2019). Cognitive Functioning in Patients with Pediatric-Onset Multiple Sclerosis, an Updated Review and Future Focus. Children.

